# Seasonal Patterns: Bovine Trypanosomosis, *Glossina pallidipes* Density, and Infection in Rift Valleys of Gamo Zone, Southern Ethiopia

**DOI:** 10.3389/fvets.2022.805564

**Published:** 2022-02-28

**Authors:** Wasihun Seyoum, Ephrem Tora, Kokeb Kore, Firew Lejebo

**Affiliations:** ^1^Department of Animal Science and Health, College of Agricultural Sciences, Arba Minch University, Arba Minch, Ethiopia; ^2^National Institute for Control and Eradication of Tsetse Fly and Trypanosomosis, Arba Minch, Ethiopia

**Keywords:** seasonal, bovine, trypanosomosis, *Glossina*, *pallidipes*

## Abstract

Bovine trypanosomosis is a parasitic disease causing serious economic losses in livestock productivity and agricultural development. The disease has been reported in different parts of Ethiopia. However, seasonal pattern of trypanosomosis, tsetse fly apparent density, and infection are very limited in the southern rift valley of the country, particularly in Gamo Zone. Therefore, the objective of this cross-sectional study design was to estimate seasonal prevalence of bovine trypanosomosis, assessing tsetse fly apparent density and its infection by trypanosomes. For the parasitological study, a total of 600 cattle (300 in each season) were sampled and assayed using the buffy coat technique. A total of 80 standard NGU traps were deployed around the watering and grazing areas for the entomological survey. An overall prevalence of trypanosomosis was 10.17% (61/600), of which 7.33% (22/300) and 13% (39/300) accounted for the dry and wet seasons, respectively. The prevalence of trypanosomosis was significantly higher during the wet season (OR = 2.47; *p* < 0.05), in black coat color (OR = 7.2, *p* < 0.05), and poor body-conditioned (OR = 3.15; *p* < 0.05) animals. Two species of trypanosomes, *Trypanosoma congolense*, 68.85% (42/61), and *Trypanosoma vivax*, 31.15% (19/61), were circulating in the area. The mean PCV value in infected animals (22.56 ± 4.61) was significantly lower than in non-infected animals (25.3 ± 4.75). Entomological result indicated that *Glossina pallidipes* (*G. pallidipes*) was the only species of tsetse found in the study area. Totally, 3,789 flies were caught of which 81.42% (3,085/3,789) belong to *G. pallidipes* and 18.58% (704/3,789) were other biting flies. The overall apparent density of *G. pallidipes* was 12.85 flies/trap/day (FTD). Relatively higher *G. pallidipes/*trap/day were caught in the wet season (13.64 F/T/D) than in the dry season (12.07F/T/D). Of the flies caught, 342 *G. pallidipes* were randomly selected and dissected. The overall proportion of *G. pallidipes* infection was 18.42% (63/342) of which 12.28% (21/171) and 24.56% (42/171) were accounted in the dry and wet seasons, respectively. Infection in *G. pallidipes* was significantly higher during the wet season (OR = 2.32; *p* < 0.05) and in park grazing areas (OR = 2.45; *p* < 0.05). In conclusion, trypanosomosis is the major challenge for cattle productivity in the district. So this study warrants the need for strengthening the vector and parasite control interventions in the area.

## Introduction

African animal trypanosomiasis is one of the major animal health problems posing a significant effect on the settlement and socioeconomic development over large tsetse belt regions of the continent. In Sub-Saharan Africa, including East Africa, the vector of a disease is distributed over 10 million km^2^ of potential grazing lands in 37 countries, exposing the lives of around 55 million people and 160 million cattle to the risk of a disease (1–4). The overall economic loss (both direct and indirect) is estimated to be about 500 billion dollars a year in terms of mortality, production, abortion, reduced fertility, and ability to work as traction animals. Furthermore, the disease is responsible for an annual loss of millions of dollars in livestock health and production as a result of the cost related to treatment, prevention, and vector control efforts (1, 5).

Ethiopia has huge livestock population in Africa, and the livestock sector plays a significant role in the national economy and livelihood of farmers and pastoralists (6). The subsector contributes about 16.5% of the national gross domestic product (GDP) and 35.6% of the agricultural GDP. Despite this huge livestock number, productivity is too low and even below the average for most Eastern and Sub-Saharan African countries, due to a number of complex and interrelated factors, such as inadequate feed and nutrition, widespread diseases, poor genetic potential of local breeds, and inefficiency of livestock development services (7). Among these, trypanosomosis is one of the major animal health constraints to livestock production and agricultural development (8).

Trypanosomosis is a chronic hemoprotozoan disease of domestic animals and humans caused by different species of unicellular eukaryotic parasite of the genus *Trypanosoma* (8). With an exception of *Trypanosoma equiperdum* of equines, which causes a venereal disease, all have arthropod vectors in which the transmission is either cyclically by tsetse flies of the *Glossina* species or non-cyclical by many other insects (9, 10). Cattle affected with trypanosomosis can show major clinical manifestations of a disease, such as intermittent fever, anemia, anorexia, dullness, apathy, watery ocular discharge, reproductive disorder, and superficial lymph node enlargement. The animals progressively become emaciated and cachectic, and finally die if untreated (11).

Bovine trypanosomosis is highly prevalent and distributed in the most arable and fertile land of the southwest and northwest part of Ethiopia following the low lands and greater river basins of Abay, Omo, Akabo, Didessa, Ghibe, and Baro (12, 13). The disease has been reported in different parts of the country with apparent prevalence ranging from 1.38 to 17.15% (14). Currently about 220,000 km^2^ areas of the abovementioned regions of the country are infested by five Glossina species, namely, *Glossina pallidipes, Glossina morsitans submorsitans, Glossina fuscipes, Glossina tachinoides*, and *Glossina longipennis*. In the country, the most commonly reported and important *Trypanosoma* species affecting cattle include *Trypanosoma congolense, Trypanosoma vivax*, and *Trypanosoma brucei* (15, 16). It is estimated that 10 to 14 million cattle heads in Ethiopia are exposed to the risk of trypanosomosis (14). Cattle production plays a key role in the livelihood of southern regions of Ethiopia, but their production potential is not fully utilized and challenged due to trypanosomosis (15, 17).

Arba Minch Zuria district of southern Ethiopia is one of the well-known tsetse belt areas of East Africa. The district was highly infested with *Glossina pallidipes* and biting flies like *Tabanus* and *Stomoxys* (18, 19). In turn, bovine trypanosomosis is one of the most important livestock diseases in the district, which poses a serious threat to the lives and livelihood of entire communities. Almost all cattle in, and adjacent of, the district are at risk of acquiring the disease at any time. As a result, people in the district suffer from low level of draft power and productivity of their animals (20–22).

Over the past few decades, many efforts have been made to control tsetse and trypanosomosis in Ethiopia through coordinated action of the government, non-governmental organizations, and local community. The control interventions commonly used in Ethiopia include insecticidal pour-on, insecticide-impregnated traps and targets, and use of different trypanocidal drugs (8, 16). However, information related to temporal and spatial dynamics of tsetse and trypanosomes remain very limited and may be a reason that control strategies are less effective and fail in endemic areas (17, 23). Hence, the epidemiological knowledge on seasonal prevalence of bovine trypanosomosis and distribution of the tsetse fly are paramount in formulating appropriate strategies for the control of these problems (24). Also in Ethiopia, a few studies were conducted regarding the seasonal dynamics on the prevalence of the disease, apparent density, and trypanosome infection rate in the tsetse fly, while no studies were performed in the current study area. Therefore, this study was conducted to estimate seasonal prevalence of bovine trypanosomosis, to assess tsetse fly apparent density and its infection by trypanosome.

## Materials and Methods

### Study Area Description

**Figure 1:**
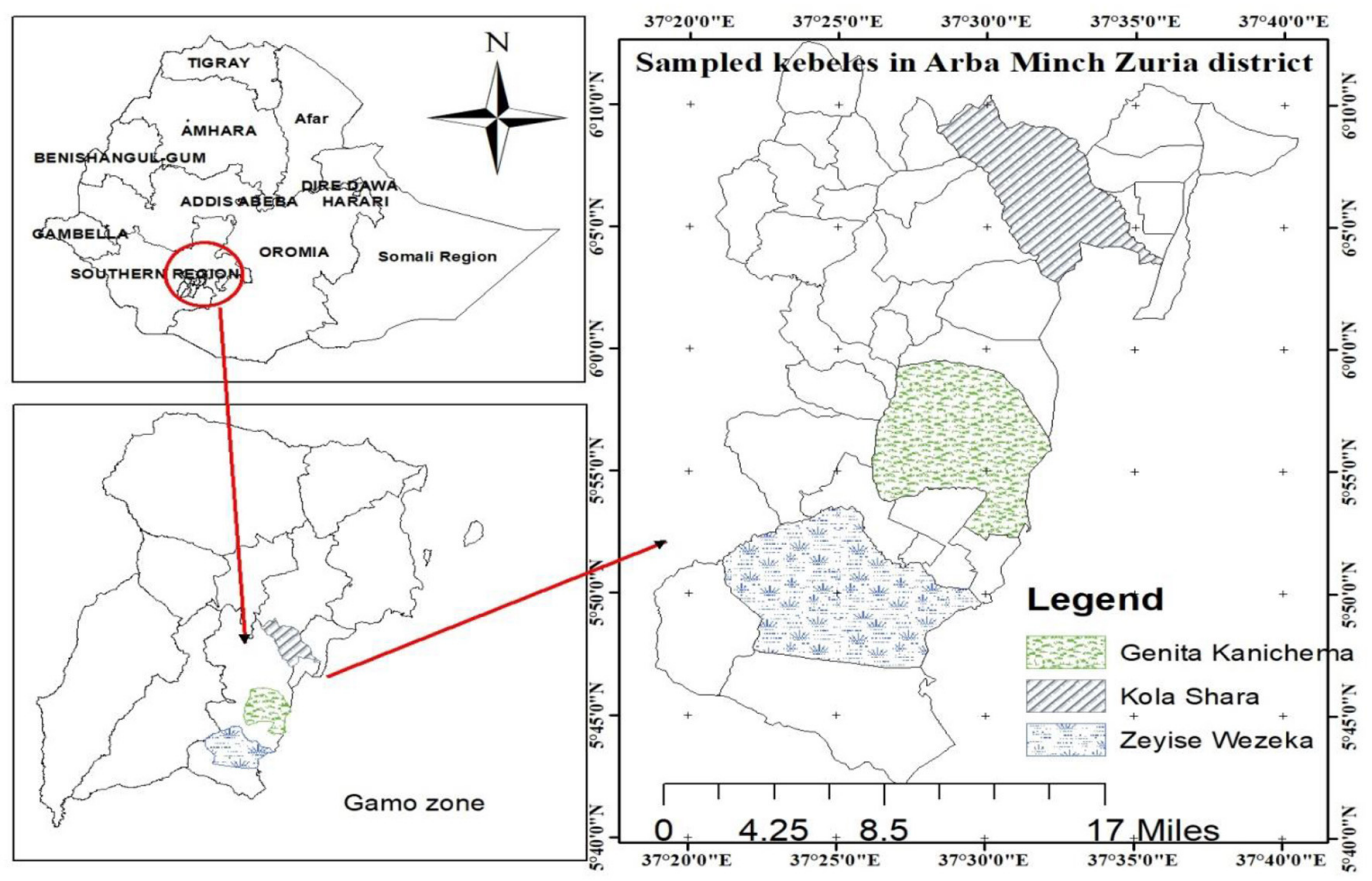
Map of the study area.

The study was conducted from July 2018 to June 2019 in the rift valley of Arba Minch Zuria district of Gamo Zone, Southern Ethiopia, which includes dry (December to February) and wet (July to September) seasons (Figure 1). The Arba Minch Zuria district is situated in the well-known area of East Africa rift valley and surrounded by the Chamo and Abaya lakes as well as the “Nech Sar” national park. Topographically, massifs, plains, steep slopes, and gorges along the course of a number of streams, rivers, and lakes mark these areas. The altitude of the area ranges from 1,001 to 2,500 masl. The area has bimodal rainfall pattern, the short rain falls between March and April, and the long rainy season between June and September. Annual rainfall ranges from 800 to 1,200 mm, and the average annual temperature is 26.33°C. The district is divided into lowland and midland agro-ecological zones, which account for about 55.55 and 45.5% of the total area, respectively. The total cattle population in the district is estimated to be 155,617. The livelihood of the society largely depends on mixed livestock and crop production (6, 25). The study area has infestation with *G. pallidipes* and biting flies like *Stomoxys* and *Tabanus* (18).

### Study Animals

The study animals used for this study were local zebu cattle (*Bos indicus*), older than 6 months of age, which were kept under an extensive management system together with other livestock species. They herded together during the day time and returned to their individual owner's farmstead each evening. The age of the study animals was categorized as young (3 years old) and adult (>3 years old) (26). According to the body condition of the animals, they were grouped as poor, medium, and good based on the appearance of ribs and dorsal spines applied for zebu cattle (27).

#### Inclusion and Exclusion

The inclusion criteria required were animals whose age were more than 6 months and managed under extensive production system. However, animals that were treated by anti-trypanocidal drugs within a month and animals age <6 months were excluded from the study. For fly dissection, trapped tsetse fly species were categorized into teneral and non-teneral. Then the teneral tsetse flies were included to precede dissection.

### Study Design

A cross-sectional study design was conducted to estimate the seasonal prevalence of trypanosomosis in cattle and the apparent density of *G. pallidipes* and its infection by trypanosome in the Arba Minch Zuria district of Southern Ethiopia.

### Sampling Method and Sample Size Determination

Purposive sampling was applied based on the complaint by animal owners on trypanosomosis to select three study kebeles from the district. Kebele refers to the lowest administrative division of a district in Ethiopia but greater than a village. The selected three kebeles were Zeyse Wezeka, Ganta Kanchama, and Kola Shara. The study animals were selected and then sampled by using the simple random sampling technique, in which all animals in the study area have equal chance of being selected. The total number of animals required for the study was computed by the formula given by Thrusfield et al. (28) using the average expected prevalence of reports, 11% in the study area (20–22), with 95% confidence interval and absolute precision of 5%. Based on this formula, the calculated sample size was 150. Then to maximize the precision, the sample size was increased by 100%. Therefore, a total of 600 animals (300 in each season) were selected for parasitological study. Potential risk factors considered in the study were season, body condition score, sex, coat color, and age of the study animals.

### Study Methodology

#### Parasitological Study

Blood samples were collected puncturing the superficial ear vein of each study animal into heparinized capillary tubes and, then after, sealed on one side with Cristaseal. Then the capillary tubes were transferred to a hematocrit centrifuge and centrifuged immediately for 5 min at 1,200 revolutions per minute. Packed cell volume (PCV) was measured by using a hematocrit reader for the determination of the level of anemia. Animals with PCV <25% were designated as anemic. The capillary tubes were then cut using a diamond pen at about 1 mm below the buffy coat to include the uppermost layers of the red blood cells and 3 mm above to include the plasma. The contents of the capillary tube were pressed onto a microscopic slide, mixed, and covered with a 22 × 22 mm cover slip. Finally, it was examined under × 40 and/or × 10 objective lens for the presence of motile trypanosomes (29). The trypanosome species were identified based on their movement pattern during the buffy coat examination as described by Murray et al. (30). The confirmation of trypanosome species by morphological characteristics was done after staining the blood smear with Giemsa and examination with oil immersion microscopy with × 100 power of magnification (8, 31).

The study protocol for the blood sampling from the ear vein was ethically reviewed and approved by the Institutional Review Board (IRB) of the Southern Ethiopia Tsetse and Trypanosomiasis Control and Eradication Institute.

#### Entomological Study

The entomological study was employed from July to September 2018 and December to February 2019, which are wet and dry seasons, respectively. A total of 80 standard Nguruman (NGU) traps (40 traps in each season) were deployed around the watering and grazing areas for the trapping of tsetse and other biting flies. The NGU trap is used to catch savanna flies, such as *Glossina pallidipes*, and is very effective and easily constructed from locally available materials (32). All traps were uniformly baited with acetone and 3-week-old cow urine deployed at an interval of 200–250 m (33). Traps were allowed to stay at the site of deployment for a maximum period of 72 h before collection (34). Trap deployment sites were selected to represent all vegetation type/habitat that could be related to fly multiplication, behavior, feeding, and other related aspects. The poles of each traps were greased to prevent fly predators, mainly ants. Then tsetse and other biting flies that were trapped were collected and counted. Tsetse flies were sexed just by observing the posterior end of the ventral aspect of the abdomen using hand lens and stereomicroscope. Hence, male flies were identified by an enlarged hypopygium in the posterior ventral part of the abdomen, which is absent in female flies. Other biting flies that were caught were identified at the genera level according to their morphological characteristics, such as size, color, wing venation structure, and proboscis (34–36). The apparent density of the tsetse fly was calculated as the number of tsetse catch/trap/day (34).

### Fly Dissection and Infection Rate Determination

Collected flies were grouped into teneral and non-teneral, and then the teneral tsetse flies were subjected to dissection and examination for infection with trypanosome (35). The dissection procedure in both study seasons was carried out as described by the FAO Training Manual for tsetse control personnel. Frist, the wings and legs of tsetse flies were removed. Wing fry analysis and ovary analysis were performed in determining the age of the male and female tsetse flies, respectively. Then 0.95% normal saline solution was used for dissecting freshly killed tsetse flies under a dissecting microscope. Three body parts of the tsetse flies, namely, the proboscis (mouth part), midgut, and salivary glands, were examined (34). A compound microscope at a magnification of × 400 times was used for the identification of trypanosome infections in the tsetse flies (37).

Trypanosome parasites detected in the mouthpart only were considered in the group of Duttonella (*T. vivax*), those detected both in the mouthparts and midguts were Nanomonas (*T. congolense*), and those found in the midgut, salivary glands, and mouthparts were considered as Trypanozoon (*T. brucei*) (17, 23, 38). Immature infections considered when the trypanosome parasites were detected only in the midgut of the tsetse flies. Finally, Giemsa-stained smears were examined under oil immersion compound microscope ( × 100 magnification) for trypanosome species identification based on morphological appearances (17, 38, 39). The infection rate (IR) in each season was calculated using the following formula (37):


$$\begin{array}{l} \text{Infectionrate}\left(\text{IR}\right)\\ =\frac{\text{Numberoftsetseflies infected}}{\text{Totalnumberoftsetsefliesdissectedoveragiven period}.} \end{array}$$

### Data Management and Statistical Analysis

Both parasitological and entomological data in each study seasons were collected and recorded in a Microsoft Excel spread sheet. The STATA version 14.2 computer software was applied for the statistical analysis at 95% confidence interval. The prevalence of trypanosomosis was calculated as the number of infected cattle divided by the total number of sampled animals and then multiplied by 100 (28). Those associated risk factors with a *p* < 0.25 in the univariable logistic analysis were included in the final multivariable logistic model. The infection rate (IR) of trypanosomes in *G. pallidipes* was calculated as the number of microscopically positive flies divided by the total number of dissected flies and multiplied by 100 (34). The apparent density (AD) of the tsetse and biting flies were expressed as the number of each type of fly per trap per day (FTD). In all cases, 95% confidence interval was used, and a *p* < 0.05 was considered as significant (40).

## Results

### Parasitological Results

#### Prevalence of Trypanosomosis

**Figure 2:**
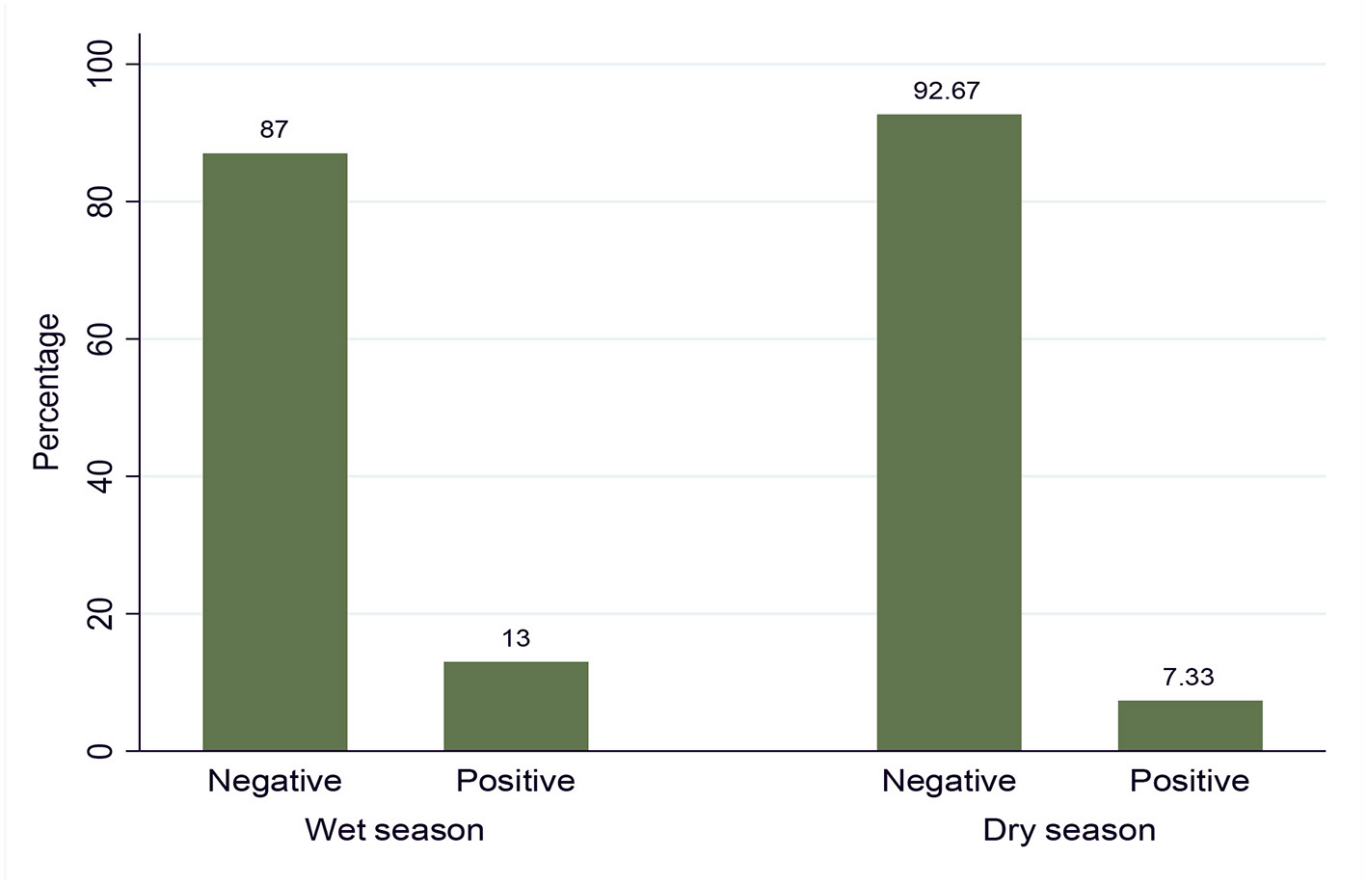
Seasonal prevalence of trypanosomosis.

Out of the total 600 examined animals by the buffy coat technique (i.e., 300 in the dry and 300 in the wet seasons), 61 animals were found to be positive for trypanosome infection, giving an overall prevalence of disease at 10.17% (61/600) in the study area. The prevalence of trypanosome infection in the dry and wet seasons is shown in Figure 2, and it was statistically significant (*p* = 0.0216).

### Proportion of Trypanosome Species Identified

Two species of trypanosomes were identified, which, in order of abundance, were *T. congolense* 68.85% (42/61) and *T. vivax* 31.15% (19/61). Infection due to *T. brucei* and mixed trypanosome species were not found in the study area during the two study seasons. The trypanosome species seasonally identified are shown in Table 1.

**Table 1:** Proportion of *Trypanosome* species identified in the dry and wet seasons (*n* = 61).

**Season**	**No. of positive animals**	***Trypanosome congolense* (%)**	***Trypanosome vivax* (%)**	**Proportion of *Trypanosome* species (%)**
Wet	39	28 (45.9)	11 (18.03)	39 (63.93)
Dry	22	14 (22.95)	8 (13.12)	22 (36.07)
Total	61	42 (68.85)	19 (31.15)	

### Analysis of Trypanosomosis With Risk Factors

The possible risk factors considered in the univariable logistic regression analysis of the presence of trypanosomosis were sex, age, coat color, body condition score (BCS), season, and kebeles of the study district. Of these factors, BCS, coat color, and season were found to be significantly (*p* < 0.05) associated with trypanosomosis, while sex, age, and kebeles of the study districts did not have a significant effect (*p* > 0.05) (Table 2). After checking for colinearity, all variables with *p* < 0.25 in the univariable analysis (BCS, coat color, and season) were subjected to stepwise backward multivariable logistic regression analysis. The final multivariable logistic regression model for potential risk factor analysis revealed that season, color, and body condition score had a significant association with trypanosome prevalence and, hence, potential factors (*p* < 0.05). The Hosmer–Lemeshow goodness-of-fit test suggested that the model fits the data (χ^2^ = 20.28; *p* = 0.0619) (Table 3).

**Table 2:** Univariable logistic regression analysis for risk factors associated with prevalence of disease.

**Variable**	**Category**	**Total animal examined**	**Number of infected animal (%)**	**OR**	**95% CI**	***p*-value**
Season	Dry	300	22 (7.33)	–	–	Ref
	Wet	300	39 (23)	1.88	1.09–3.27	0.023
Sex	Male	246	24 (9.75)	–	–	Ref
	Female	354	37 (10.45)	1.07	0.62–1.85	0.782
Age	Adult	330	31 (9.39)	–	–	Ref
	Young	270	30 (11.11)	1.205	0.71–2.04	0.489
Coat color	White	215	11 (5.11)	–	–	Ref
	Red	251	14 (5.57)	1.33	0.50–3.56	0.559
	Black	134	36 (26.86)	8.32	3.37–20.52	0.001
BCS	Good	175	10 (5.71)	–	–	Ref
	Medium	207	14 (6.76)	1.19	0.51–2.76	0.674
	Poor	218	37 (16.97)	3.37	1.62–6.99	0.001
Kebeles	Kola Shara	200	17 (8.5)	–	–	Ref
	Ganta Kanchama	200	26 (23)	1.60	0.84–3.06	0.149
	Zeyse Wezeka	200	18 (14)	1.06	0.53–2.13	0.86

**Table 3:** Multivariable logistic regression analysis of the association of trypanosomosis with potential risk factors.

**Variable**	**Category**	**Total animal examined**	**Number of infected animals (%)**	**OR**	**95% CI**	***p*-value**
Season	Dry	300	22 (7.33)	–	–	Ref
	Wet	300	39 (23)	2.47	1.35–4.49	0.003
Color	White	142	11 (5.11)	–	–	Ref
	Red	251	14 (5.57)	1.17	0.52–2.67	0.701
	Black	134	36 (26.86)	7.2	3.62–16.05	0.001
BCS	Good	175	10 (5.71)	–	–	Ref
	Medium	207	14 (6.76)	1.27	0.53–3.04	0.58
	Poor	218	37 (16.97)	3.15	1.45–6.74	0.004

### Hematological Findings

The hematological findings revealed that overall mean (±SD) PCV value of all studied animals was 25.02% (±4.8) (95% CI = 24.64–25.41) (Table 4). The mean PCV of infected animals (22.56 ± 4.61) was significantly (*p* < 0.001) lower than that of the non-infected ones (25.3 ± 4.75%). Moreover, the mean PCV of animals in the wet season (24.85 ± 4.77) was lower than that of the animals in the dry season (25.2 ± 4.84) with no significant difference (*p* > 0.05).

**Table 4:** Analysis of the association of trypanosome infections with mean packed cell volume (PCV) (%) of cattle.

**Variable**	**Category**	**Animal examined**	**Mean PCV (%)**	**Std. dev**.	**95% CI**	**t-test**	***p*-value**
Infection status	Non-infected	539	25.3	4.75	24.9–25.71		
	Infected	61	22.56	4.61	21.38–23.74	4.27	0.001
Season	Dry	300	25.2	4.84	24.65–25.74		
	Wet	300	24.85	4.77	24.31–25.39	0.89	0.1863
	Overall	600	25.02	4.8	24.64–25.41		

**Figure 3:**
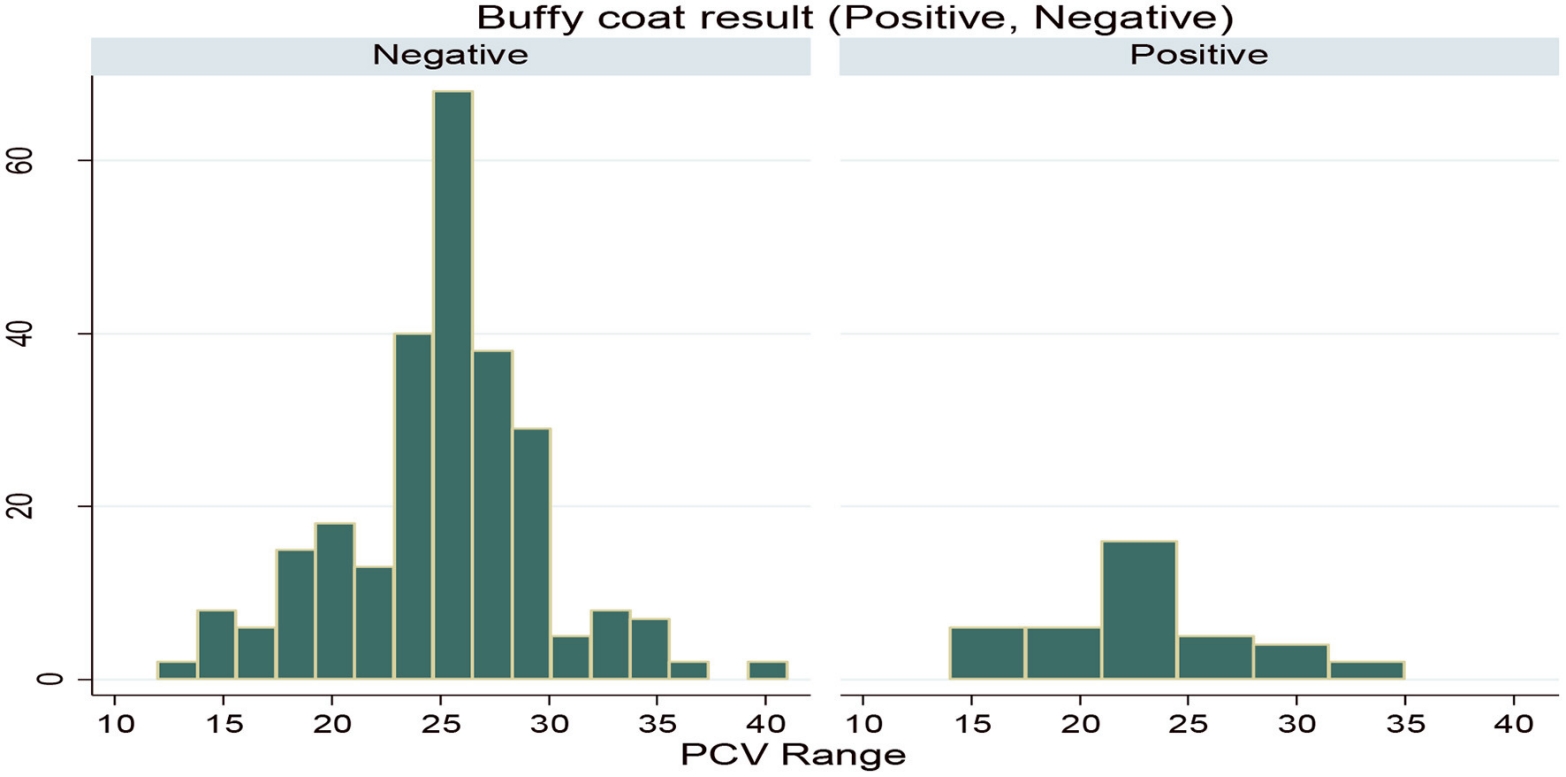
Packed cell volume (PCV) distribution for infected and non-infected animals.

During the study period, a total of 322 (53.66%) animals were between the PCV range of 12 and 25, which was anemic, while the rest of the 278 (46.33%) animals were between the PCV range of 25 and 41, which were non-anemic. Also, based on the PCV category, the prevalence in the anemic animals (16.67%) was higher than the prevalence in the non-anemic animals (5.7%). PCV distribution among the infected and non-infected animals during the study period is indicated in Figure 3.

### Entomological Results

#### Seasonal Proportion of Fly Count

A total of 3,789 tsetse and other biting flies were caught with 80 NGU traps deployed in the study area. Out of these, 81.42% (3,085/3,789) belong to tsetse flies and the remaining 18.58% (704/3,789) were biting flies. *G. pallidipes* was identified as the only tsetse fly species in the study area. The biting flies that were commonly encountered during the study period were the *Stomoxys* species (45.17%, 318/704) and *Tabanus* species (54.82%, 386/704). The result of the total fly count showed that a high proportion of the flies was counted in the wet season than in the dry season. A total of 53.94% (2,044/3,789) of the flies were caught during the wet season, and 46.05% (1,745/3,789) of the flies were caught during the dry season. Also, the results for each fly type count indicated that there was a high proportion of fly count in the wet season than in the dry season (Table 5).

**Table 5:** Proportion of each fly count in the wet and dry seasons.

**Season**	**Tsetse fly no. (%)**			**Biting fly no. (%)**			**Total fly count**
	**Male**	**Female**	**Total**	***Tabanus***	***Stomoxys***	**Total**	
Wet	531 (32.45)	1,105 (67.5)	1,636	221 (54.16)	187 (45.83)	408	2,044
Dry	328 (22.63)	1,121 (77.36)	1,449	165 (55.74)	131 (44.25)	296	1,745
Total	859 (27.84)	2,226 (72.15)	3,085	386 (54.82)	318 (45.17)	704	3,789

### Seasonal Apparent Density

The overall apparent densities of *G. pallidipes* and biting flies in the study area were 12.85 F/T/D (fly/trap/day) and 2.93 F/T/D, respectively. The apparent density of *G. pallidipes* and biting flies was higher in the wet season than in the dry study season (Table 6). It was found to be 13.63 F/T/D and 3.4 F/T/D, respectively, for the tsetse and biting flies in the wet season and 12.07 F/T/D and 2.46 F/T/D, respectively, for the tsetse and biting flies in the dry season with a significant difference (*p* < 0.05). Based on the kebeles, in both study seasons, a high apparent density of tsetse and biting flies was found in and around the Nech Sar national park compared with the communal grazing areas of Zeyse Wezeka, Ganta Kanchama, and Kola Shara kebeles of the study area.

**Table 6:** Seasonal apparent density of *Glossina pallidipes* and biting flies in the study area.

**Season**	**Kebeles**	**No. of traps used**	***G. pallidipes***			**Biting flies**		
			**Male**	**Female**	**Total**	**F/t/d**	**Count**	**F/t/d**
Wet	Zeyse Wezeka	10	91	155	246	8.2	108	3.6
	Ganta Kanchama	10	154	287	441	14.7	51	1.7
	Kola Shara	10	70	127	197	6.5	67	2.23
	In and around Nech Sar park	10	216	536	752	25.06	182	6.07
	Total	40	531	1,105	1,636	13.63	408	3.4
Dry	Zeyse Wezeka	10	72	124	196	6.53	59	1.96
	Ganta Kanchama	10	85	301	386	12.8	51	1.7
	Kola Shara	10	61	106	167	5.56	38	1.26
	In and around Nech Sar park	10	110	590	700	23.33	148	4.93
	Total	40	328	1,121	1,449	12.07	296	2.46
	Season total	80	859	2,226	3,085	12.85	704	2.93

**Figure 4:**
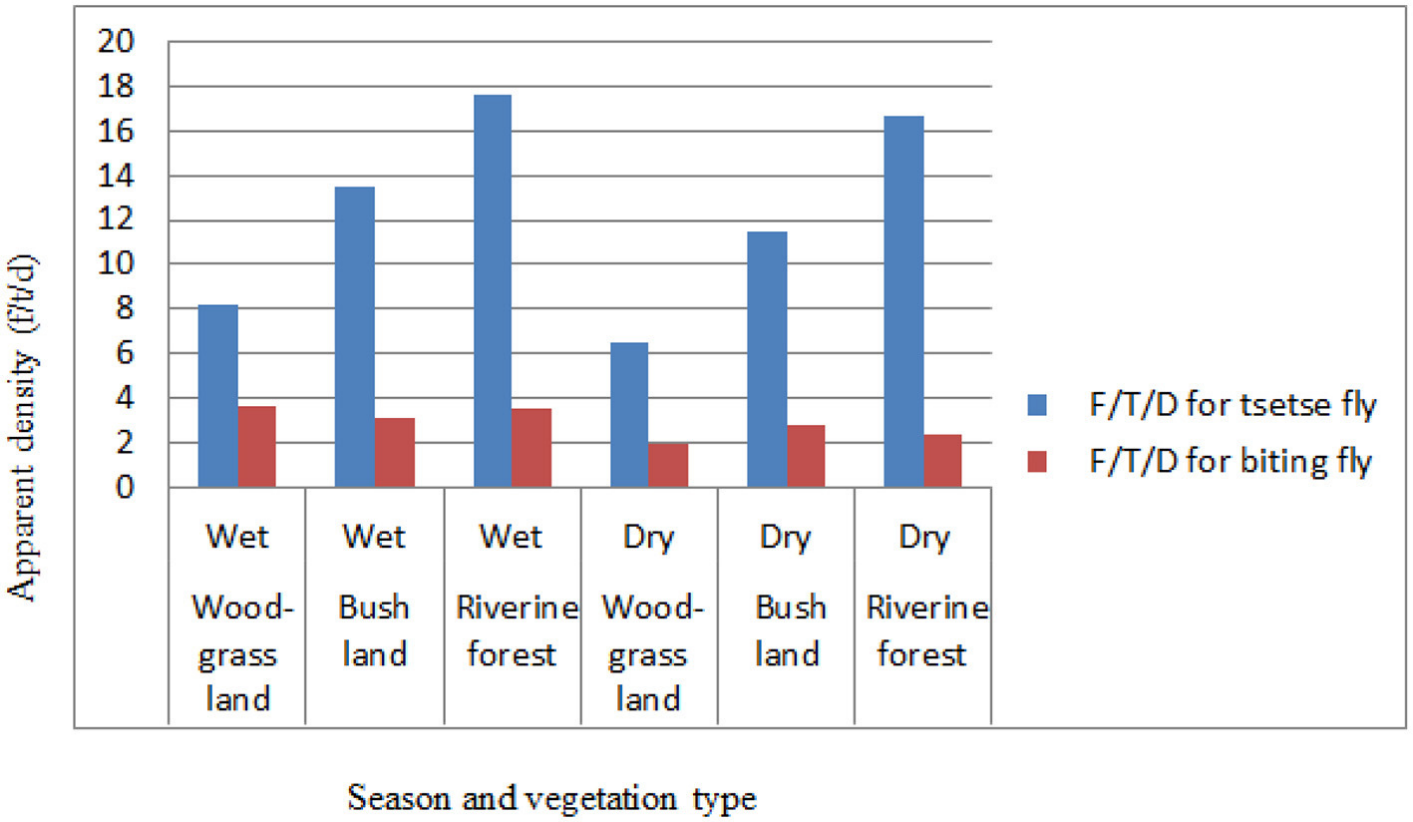
Seasonal apparent density based on vegetation.

Based on the vegetation type in the study area, relatively the apparent density of the tsetse flies was higher in the riverine forest than in the wood grassland (WGL) and bushland vegetation types both in the wet and dry seasons of the study period. The apparent densities for tsetse flies were 8.2, 13.54, and 17.6, respectively, for wood grassland, bushland, and riverine forest in the wet season and 6.53, 11.47, and 16.71, respectively, for wood grassland, bushland, and riverine forest in the dry season of the study period. The apparent densities of biting flies were relatively high in the wood grassland in the wet season and bushland vegetation types in the dry season of the study period. The apparent densities for biting flies were 3.6, 3.16, and 3.52, respectively, for wood grassland, bushland, and riverine forest in the wet season and 1.96, 2.81, and 2.42, respectively, for wood grassland, bushland, and riverine forest in the dry season of the study period (Figure 4).

### Trypanosome Infection Rate in *Glossina pallidipes*

From a total of 342 dissected tsetse flies (*G. pallidipes*), 63 flies were infected with trypanosome resulting in an overall infection rate of 18.42% (63/342) in the study area. High trypanosome infections were observed in the wet season (24.56%, 42/171) than in the dry season (12.28%, 21/171) of the study periods. *T. congolense* (74.6%, 47/63) was the predominant species and found to be a major cause of tsetse fly infection in the study area followed by *T. vivax* (25.39%, 16/63). Infection of *G. pallidipes* due *T. brucei* and mixed type was not found in the study area during the two study seasons (Table 7).

**Table 7:** Seasonal infection rate in *Glossina pallidipes* and identified trypanosome species.

**Season**	**No. of flies dissected**	**No. of infected flies**	***T. congolense (%)***	***T. vivax (%)***	**Overall infection (%)**
Wet	171	42	31 (73.81)	11 (26.19)	42 (24.56)
Dry	171	21	16 (76.19)	5 (23.81)	21 (12.28)
Total	342	63	47 (74.603)	16 (25.39)	63 (18.42)

Trypanosome infection in *G. pallidipes* and its association with potential risk factors are summarized in the univariable logistic regression analysis (Table 8). This result showed that the infection rate in *G. pallidipes* during the wet season was 2.32 higher than in the dry season. Also, trypanosome infection in *G. pallidipes* in and around the park grazing area was 2.45 significantly higher than communal grazing areas. Infection rate based on vegetation type, kebele, sex, and age categories of flies did not show a significant difference (*p* > 0.05).

**Table 8:** Univariable logistic regression analysis for potential risk factors of trypanosome infection in *G. pallidipes*.

**Variable**	**Category**	**No flies examined**	**No flies infected**	**Infection rate (%)**	**OR**	**95% CI**	***p*-value**
Season	Dry	171	21	12.28	–	–	Ref
	Wet	171	42	24.56	2.32	1.30–4.12	0.004
Grazing area	Communal	192	24	12.5	–	–	Ref
	Park area	150	39	26	2.45	1.40–4.31	0.002
Sex	Male	127	20	15.75	–	–	Ref
	Female	215	43	20	1.33	0.746–2.39	0.328
Age	≤ 20 days	49	4	8.16	–	–	Ref
	>20 days	293	59	20.13	2.83	0.981–8.20	0.054
Vegetation type	WGL	50	5	10	–	–	Ref
	Bush land	131	22	16.79	1.82	0.64–5.09	0.257
	Riverine forest	161	36	22.36	2.59	0.95–7.01	0.061
Kebeles	Kola Shara	50	4	8	–	–	Ref
	Nech Sar park	150	39	26	4.04	1.36–11.95	0.012
	Ganta Kanchama	92	15	16.3	2.24	0.70–7.15	0.174
	Zeyse Wezeka	50	5	10	1.27	0.32–5.06	0.727

## Discussion

The present study revealed that trypanosomosis is an important disease of cattle in the rift valley area, Arba Minch Zuria district, Southern Ethiopia, with an overall prevalence of 10.17%. This result was higher than the findings of Teka et al. (20), Girma et al. (41), and Anjulo et al. (22) who reported an overall prevalence of 4.1, 1.56, and 1.75%, respectively, in the current study area. In contrast to this, Zeryehun and Abraham (21) reported a higher prevalence of disease (27.5%) in the study area. Similarly, higher prevalence estimates were reported in Refs. (42–44) from different parts of Ethiopia. Likewise, studies in the Kilwa district of southern Tanzania and in Kaduna State of Central Nigeria showed overall prevalence of 9.3 and 9.13%, respectively (45, 46). However, studies reported by Mbahin et al. (47) in Kwale county of Coastal Kenya and Kizza et al. (48) in Murchison Falls National Park of Uganda showed higher overall prevalence of 33.9 and 29.6%, respectively. Such variation between reports might be due to the differences in the season of the study period, management system, vector infection rate, animal susceptibility, trypanocidal drug usage, fly control operations, increase of tsetse challenges, and awareness level of the animal owners about the disease in a given study areas (44, 49).

Two species of trypanosomes were identified in the current study areas, namely, *T. congolense* (68.85%) and *T. vivax* (31.15%). This study has confirmed the observation of (8, 13, 16, 17, 49) in different parts of Ethiopia. The relatively higher predominance of *T. congolense* infection in cattle suggests the increased contact of cattle with the savanna tsetse flies, particularly *G. pallidipes*, which are more efficient transmitters of *T. congolense* than *T. vivax* in East Africa (12, 50). It may be also due to the high number of serodems of *T. congolense* compared with *T. vivax* and the development of better immune response to *T. vivax* by the infected animal in the study area (51).

The multivariable analysis for potential risk factors showed significantly higher prevalence of trypanosomosis in the wet season (OR = 2.47, *p* < 0.05), black coated (OR = 9.3, *p* < 0.05), and poor body-conditioned animals (OR = 3.5, *p* < 0.05). This result was in close agreement with the previous study reported in Refs. (17, 42, 44). In the current study, seasonal prevalence of trypanosomosis was higher in the wet season (13%) than in the dry season (7.33%). This may support the statement that season is a well-known limiting factor for cattle trypanosomosis (23). This might be due to an absolute increase in the number of tsetse and biting flies in the wet season due to favorable environmental factors, such as enough moisture, vegetation growth, and suitable habitat, which, in turn, lead to an increase in trypanosome challenge to cattle resulting into the observed difference during the two study seasons (33, 42).

Black-coated animals were more affected by trypanosomosis than the other color in the study area. This is because of the existing knowledge that *Glossina* species are more attracted to large dark colors like the hides of cow (52). Besides, trypanosomosis was observed to be higher in animals with poor body condition when compared with those in good or medium body conditions. It meant that trypanosomosis causes emaciation of animals (8, 20). Although poor body condition could result from other factors, such as concurrent nutritional and parasitic diseases, bovine trypanosomosis is also a devastating and wasting disease, which results in a progressive loss of body condition (53).

The overall mean PCV value of all studied animals was 25.02%. The mean PCV of animals in the wet season (24.85 ± 4.77) was lower than that of the animals in the dry study season (25.2 ± 4.84), which is similar to the results obtained in Refs. (17, 42, 44). The mean PCV of the infected animals (22.56 ± 4.61) was significantly (*p* < 0.001) lower than that of the non-infected ones (25.3 ± 4.75%). The lower mean PCV value in the infected animals have been reported by authors from different corners of the country (21, 22, 39, 43, 49). Variation between infected and non-infected animal mean PCV value indicates that trypanosomosis might be involved in the reducing of the PCV values in the infected animals (30). However, trypanosomosis is typically suspected to reduce the PCV. It is worthy to note that other factors like blood-sucking gastrointestinal parasites and nutritional deficiencies also cause the lowering of PCV (17, 40).

Furthermore, the overall occurrence of anemia in animals examined for PCV value was 53.66% in the study area, and the presence of anemia was higher in trypanosome-positive animals (16.67%) than in negative animals (5.7%). This finding agreed with previous reports done by Desta et al. (39) in the nearby Amaro special district, Eyasu et al. (17) in the escarpment of the Omo River, Loma district of Southern Ethiopia, Biyazen et al. (13) in the Dale Wabera district of Kellem Wollega Zone of Western Ethiopia, Abebe et al. (8) in the Omo-Ghibe tsetse belt areas of South Ethiopia, and Yalew and Fantahun (43) in the Bambasi district of Western Ethiopia. This is because anemia is recognized and considered as the most important pathological consequence of African animal trypanosomosis (40).

In this study. entomological findings revealed the presence of only one *Glossina* species, known as *G. pallidipes*, and other biting flies including *Stomoxys* and *Tabanus*. A total of 3,085 (81.42%) *G. pallidipes* and 704 (18.58%) other biting flies were caught during the study period. The overall apparent densities of *G. pallidipes* and biting flies in the study area were 12.85 F/T/D (fly/trap/day) and 2.93 F/T/D, respectively, which were lower when compared with previous reports by Teka et al. (20) and Rodrigues et al. (19) with an overall apparent densities of *G. pallidipes* of 29.624 F/T/D and 47.8 F/T/D, respectively, in the study area. However, the reports done in Refs. (8, 17, 22, 38, 43, 54) in different parts of Ethiopia showed a low apparent density of tsetse and other biting flies. These variations are due to differences in study seasons, vegetation type, availability of host animals, and control strategies applied in the respective places (23, 33, 51).

An overall apparent density of *G. pallidipes* in the wet season (13.63 F/T/D) was higher compared with that in the dry season (12.07 F/T/D), similar to the results obtained Refs. (17, 42, 44) in different parts of the country. This could suggest an absolute increase in the number of tsetse and biting flies due to favorable environmental conditions (33). In streak to this study, Nthiwa et al. (55) reported a higher apparent density of *G. pallidipes* in the Mtito Andei Division of Kenya, whereas Nnko et al. (23) in the Maasai Steppe of Tanzania and Bouyer et al. (56) in the Mouhoun River of Burkina Faso reported a lower apparent density number of *G. pallidipes*. This variation might be due to differences in seasons, vegetation type, availability of host animals, and control strategies.

The different habitats of vegetation were assessed during the entomological survey period, and there was a variation in the apparent density distribution of tsetse and biting flies in three vegetation types in the study area. Relatively higher numbers *G. pallidipes* and biting flies were caught during the wet season in all vegetation types, while most tsetse populations were captured in riverine forest than in wood grassland and bushland vegetation types. In line with this result, Desta et al. (54) and Dagnachew et al. (42) showed a high apparent density of tsetse flies in the riverine vegetation type followed by savanna, forest, bush, and cultivated areas. The observed variation is in close agreement with the study of Riordan (57) in which flies of the *morsitans* group (subgenus *Glossina* species) inhabits mainly riverine forest edges and savanna woodland. A similar finding was reported by Ouma et al. (58), Cecchi et al. (59), and Nthiwa et al. (55) in other eastern African countries, such as Uganda, Somalia, and Kenya, respectively. This is because open savanna woodland and riverine forest edges are a typical habitat for the *Glossina morsitans* group (mainly for *G. pallidipes*) (37, 59).

Out of the 342 dissected *G. pallidipes*, 63 flies were infected by trypanosome resulting in an overall infection rate of 18.42% in the study area. In comparison with this finding, the report of Rodrigues et al. (19) showed a higher trypanosome infection rate (38%) in *G. pallidipes* inside Nech Sar national park of Arba Minch Zuria. Also, lower infection rates were reported by comparable studies in the Mtito Andei Division in Kenya and the Mouhoun River in Burkina Faso, which show the overall trypanosome rates of 10 and 11.53%, respectively (55, 56). In contrast to these, studies in the Maasai Steppe in Tanzania and Adamawa region of Cameroon reported overall trypanosome infection rates of 5.8 and 0.9%, respectively (23, 60). Desta et al. (39) and Meharenet and Alemu (38) reported that a relatively low fly infection rate was observed in different parts the country. This might be due to the least tsetse challenge, variation in *Glossina* species involved, and low fly animal contact during the study period (61).

*T. congolense* (74.6%) was the predominant species and found to be a major cause of tsetse fly infection in the study area followed by *T. vivax* (25.39%). Likewise, different authors across Africa reported that *T. congolense* is the leading species for tsetse fly infection (55, 56, 60). Moreover, Langridge (50) and Abebe and Jobre (12) stated that *T. congolense* is one of the important mouthparts and midgut trypanosome parasites because of its pathogenicity to cattle and its relatively higher infection rate in *G. pallidipes* which was completely supported by the present findings.

A higher trypanosome infection rate in *G. pallidipes* was observed in the wet season (24.56%) than in the dry season (12.28%). This is evidenced by univariable analysis for statistical significance as risk factor that showed a significantly higher infection rate in the wet season (OR = 2.32, *p* < 0.05) and nearby park grazing area of the study (OR = 2.45, *p* < 0.05). This result was similar with previous reports done in Refs. (17, 42, 54). The similarity could be due to an absolute increment both in disease prevalence and number of tsetse and biting flies due to favorable environmental conditions (33, 42). Trypanosome infection rate of *G. pallidipes* in and around the park grazing area (26%) was significantly (*p* < 0.05) higher than in communal grazing areas (12.5%). The presence of high trypanosome infection rate in park grazing areas could be associated with factors, such as availability of different vegetation types like bushland and riverine forest, which create suitable conditions for growth and development of tsetse flies, presence of many wild animals as trypanosome reservoirs in areas close to the park, which, in turn, increases contact with vectors and control programs applied (23, 51). According to Majekodunmi et al. (62) and Eyasu et al. (17), a positive relationship was observed between seasonal trypanosome prevalence, *Glossina* species apparent density, and infection rate, which were completely supported by the present study.

The present study was conducted to estimate the prevalence and infection rate of trypanosomes. Although the study was limited for the parasite detection techniques like buffy coat and Giemsa stain, molecular techniques have been shown to provide a much more accurate and complete picture of the prevalence and taxonomic level of *trypanosomal* infections in tsetse flies.

## Conclusion

This study indicated that trypanosomosis is an important disease and a potential threat to health and productivity of cattle in Arba Minch Zuria, Southern Ethiopia. A parasitological study revealed the overall prevalence of 10.17% in the study area with seasonal prevalence of 7.33% and 13% in the dry and wet seasons, respectively. Two pathogenic species, *T. congolense* and *T. vivax*, were responsible for the disease in the study area. Significant difference in prevalence was observed between seasons, coat color, and animal body condition scores. The entomological findings revealed the presence of only one *Glossina* species, known as *G. pallidipes*, and other biting flies, including *Stomoxys* and *Tabanus*. The overall apparent densities of *G. pallidipes* and biting flies in the study were 12.85 F/T/D and 2.93 F/T/D, respectively. A relatively higher *Glossina*/trap/day was caught in the wet season and in riverine forest vegetation type. The overall trypanosome infection rate in *G. pallidipes* was 18.42% with a higher proportion of infection in the wet season (24.56%) than in the dry (12.28%) season. Moreover, this study showed a direct relationship between seasonal trypanosomosis prevalence, *G. pallidipes* apparent density, and its infection by trypanosome. Hence, this study warrants the need for strengthening the vector and parasite control interventions in the area.

## Data Availability Statement

The original contributions presented in the study are included in the article/Supplementary Materials, further inquiries can be directed to the corresponding author.

## Ethics Statement

The animal study was reviewed and approved by Animal Research Ethics Review Committee of Southern Ethiopia Tsetse and Trypanosomiasis Control and Eradication Institute. Written informed consent was obtained from the owners for the participation of their animals in this study.

## Author Contributions

WS and ET conceived and designed the study, drafted the manuscript, and analyzed and interpreted the data. WS, KK, and FL performed the field and lab works. All authors read, revised, and approved the final version of the manuscript for the publication.

## Conflict of Interest

The authors declare that the research was conducted in the absence of any commercial or financial relationships that could be construed as a potential conflict ofinterest.

## Publisher's Note

All claims expressed in this article are solely those of the authors and do not necessarily represent those of their affiliated organizations, or those of the publisher, the editors and the reviewers. Any product that may be evaluated in this article, or claim that may be made by its manufacturer, is not guaranteed or endorsed by the publisher.
